# LDH-C can be differentially expressed during fermentation of CHO cells

**DOI:** 10.1186/1753-6561-5-S8-P107

**Published:** 2011-11-22

**Authors:** Berthold Szperalski, Christine Jung, Zhixin Shao, Anne Kantardjieff, Wei-Shou Hu

**Affiliations:** 1 Pharma Biotech, Roche Diagnostics GmbH, 82377 Penzberg, Germany; 2 University of Minnesota, Minneapolis, MN 55455, USA; 3 Alexion Pharmaceuticals, Cheshire, CT 06410, USA

## Abstract

Expression of CHO mRNA was measured with special microarrays from the Consortium for Chinese Hamster Ovary (CHO) Cell Genomics led by Prof. Wei-Shou Hu of the University of Minnesota and Prof. Miranda Yap of the Bioprocess Technology Institute of A*STAR, Singapore

(http://hugroup.cems.umn.edu/CHO/cho_index.html). Cultivation experiments were performed in small scale 2L stirred tank bioreactors. During fermentation a temperature shift of -3°C was performed. This was accompanied by a reduction of the cell specific lactate production rate.

The analysis of transcriptome samples before and after the temperature shift with microarrays showed several changes in the expression of available gene markers. LDH-C expression raised about 2 fold after temperature shift. LDH-A did not change. As LDH-C is known to be a specialized isoenzyme in sperm cells for consuming lactate in a lactate containing milieu, LDH-C could be proposed as a target for genetic engineering, facilitating lactate consumption in the late phase of high cell density cultures and prolonging longevity of CHO production cultures by reducing lactate and base accumulation.

## Methods

CHO-cells producing a recombinant human antibody were cultivated in a proprietary proteinfree medium and inoculated in 4 x 2L stirred tank bioreactors. Bioreactors were controlling pH, pO2 and temperature. A fixed feeding protocol was used to overcome the limitation of consumed medium components. Temperatures of 2 cultures were shifted at day 4 from 37°C to 34°C. Daily samplings of the cultures were performed to monitor cell density and viability by using an automated **Cedex™** cell counter and the trypan blue exclusion method. The supernatant of the culture was monitored for product concentration, glucose, glutamine, lactate, ammonium. Measurement of LDH (lactate dehydrogenase ) in cell culture supernatant was used as an indicator of cell lysis. Sedimented cells of cell culture samples were prepared and cRNA was processed according to **Affymetrix™** standard procedures.[1] and hybridized with custom CHO **Affymetrix™** arrays from the Consortium for Chinese Hamster Ovary (CHO) Cell Genomics [2].

## Results

**Figure 1:**
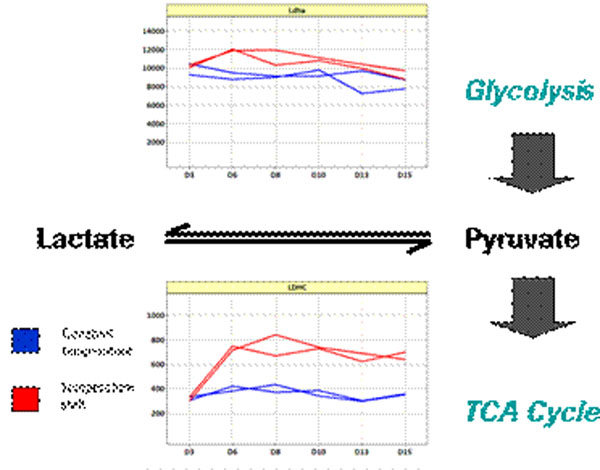


The comparison of temperature shifted and control cultures showed significant differences in the growth curves of the experiment. Temperature shift induced an early shift to the plateau phase. It reduced the cell death. Cell specific productivity was slightly higher. Lactate consumption was higher and started earlier than in control cultures (data not shown). PCA (principal component analysis) was used to compare expression ratios at different temperatures. PC 1 showed that most expression changes are onset at day 6 and maintained throughout the rest of the culture. Transcriptome analyses showed several significant changes after the temperature shift (Table 1). One outstanding result is the upregulated RNA of LDH-C (Figure 1). LDH-A RNA expression showed no significant change after temperature shift.

**Table 1:** 

Correlation to PC	Gene set	Number of genes in gene set	Nominal p-value
Positive to PC 1	Cell cycle	26	0
	DNA replication	24	0
	Cytoskeleton	67	0.02
	Microtubule organizing center	24	0.04

Negative to PC 1	Golgi apparatus	50	0
	Cell-cell signaling	48	0

Positive to PC 2	RNA processing	43	0.01
	Proteolysis	46	0.05

Negative to PC 2	DNA replication	32	0.04

## Discussion

LDH-C is known to be present in sperm cells , testis cells and some tumors [3] but is not reported to be regulated in CHO-cell lines. In sperm cells LDH-C is known to have different kinetic properties compared to A and B isoforms of LDH preferring lactate as substrate [4]. LDH-C is localized in cytoplasm and in specific “sperm type mitochondria” and seems to be integrated in a shuttle system for the transfer of reducing activity into the mitochondrial matrix [7][8]. An pseudogene association with mitochondrial cyclophilin D is reported in the gene bank of mouse genome [9]. The role of LDH-C in CHO-Cells is still unclear. The influence of temperature shift under normal body temperature seems to induce a special situation for sperm cell migration. LDH-C helps sperm cells to survive in lactic acid containing micro milieus of the oviduct. It allows lactic acid to be an energy source. These functions could be mimicked in a high lactate containing, temperature shifted fermentation process with CHO cells. LDH-C can also be regulated by hormonal mechanisms. They are known to have slight regulatory influence on the transcriptional expression [5]. Selective inhibitors of LDH isoforms are described [6]. Specific inhibitors for LDH -C are proposed as antifertilizing drugs [6]. Inhibitors to LDH-A and -B could help to favor LDH-C and so reduce lactate production. LDH-C is an interesting target for engineering manufacturing processes with cell lines like CHO cells for shifting these cells to aerobic lactate metabolism and improving growth performance.
